# Localized Lead‐Chelating Insulator Bottom Contact for Efficient and Stable p‐i‐n Perovskite Solar Cells

**DOI:** 10.1002/advs.202509816

**Published:** 2025-07-18

**Authors:** Ying Li, Yan Chen, Kang Ding, Yuanyuan Guo, Yuhan Liu, Shiwei Lu, QingQing Zou, Rongzhou Liang, Sijin Liu, Zhimin Liu, Haipeng Xie, Dongsheng Tang, Le‐Man Kuang, Yaxin Zhai, Jifei Wang

**Affiliations:** ^1^ Key Laboratory of Low‐Dimensional Quantum Structures and Quantum Control of Ministry of Education Institute of Interdisciplinary Studies Department of Physics Hunan Normal University Changsha 410081 China; ^2^ Hunan Research Center of the Basic Discipline for Quantum Effects and Quantum Technologies Hunan Normal University Changsha 410081 China; ^3^ Key Laboratory of Multifunctional Ionic Electronic Materials and Devices School of Physics and Electronics Hunan Normal University Changsha 410081 China; ^4^ Institute of Flexible Electronics (IFE) Northwestern Polytechnical University Xi'an Shanxi 710072 China; ^5^ School of Science East China Jiaotong University Nanchang 330013 China; ^6^ Hunan Key Laboratory of Super Microstructure and Ultrafast Process School of Physics and Electronics Central South University Changsha Hunan 410083 China

**Keywords:** buried interfaces, crystal facet, hole‐transport materials, perovskite solar cells, stability

## Abstract

The defective bottom interfaces of perovskites and hole‐transport layers (HTLs) limit the performance of p‐i‐n structure perovskite solar cells (PSCs). Here, that adding an ultrathin local contact layer of lead chelation polymer poly (methyl methacrylate) (PMMA) on PTAA HTLs is reported to fill its pinholes and strongly coordinate with the bottom uncoordinated Pb^2+^ simultaneously, resulting in a reduced amorphous region in perovskites near HTLs and a passivated perovskite bottom surface. Transient reflection (TR) spectroscopy results show that the ultrathin insulation layer does not affect the charge transport and collection efficiency at the PTAA and perovskite interface. This locally contacted PMMA can trigger a template‐induced specific facet orientation growth, regulating the (111) facet as the preferred facet orientation and releasing the residual lattice strain for perovskites. In addition, the deep HOMO energy of PMMA can alleviate the permeation of I_2_ into PTAA and suppress the reaction between them. Therefore, a performance of 22.6% and much‐enhanced stability (e.g., stability of T_80_ for photo‐oxygen increased over 5 times, light‐thermal over 4 times) of FA_0.90_Cs_0.10_PbI_2.83_Br_0.17_‐based PSCs is demonstrated.

## Graphical abstract

An insulating polymer, PMMA, is introduced to reinforce the heterointerfaces between perovskite and PTAA hole‐transport‐layer (HTL). It mitigated the buried interface defects, regulated the residual lattice strain, and facilitated the (111) facet as the preferred facet orientation in FA_0.90_Cs_0.10_PbI_2.83_Br_0.17_‐based perovskites. Finally, an enhanced PCE and long‐term operational stability of FA_0.90_Cs_0.10_PbI_2.83_Br_0.17_‐based PSCs is demonstrated.

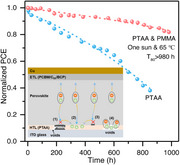

## 1 Introduction

Advancing inverted (p‐i‐n) perovskite solar cells (PSCs) is key to further enhancing the power conversion efficiency (PCE) and stability of flexible and perovskite‐based tandem photovoltaics.^[^ ^1^, ^2^, ^3^ ^]^ The defective interface‐induced carrier recombination is one of the dominant loss mechanisms in high‐efficiency PSCs, and has also been linked to hysteresis and operational stability. In inverted PSCs, the perovskite absorber is deposited on a hole‐transport layer (HTL), which plays an important role in perovskite nucleation and heterojunction formation.^[^ ^4^, ^5^ ^]^ However, the commonly used HTLs, such as polytriarylamine (PTAA), NiOx, PEDOT:PSS, have insufficient hole selectivity and/or electron trapping at the HTL/perovskite or ITO/HTL interface. Though the newly explored self‐assembled monolayers (SAM) HTL, such as the organic molecule features a phosphonic acid group (e.g., MeO‐2PACz or MeO‐4PACz),^[^ ^6^, ^7^, ^8^, ^9^ ^]^ have made great breakthroughs in improving the efficiency and stability of PSCs. Still, the study also proved that PSCs based on these SAM HTLs rapidly decomposed under reverse bias^[^ ^10^ ^]^ and operated in MPPT.^[^ ^11^ ^]^ Fortunately, the success of p‐i‐n structured PSCs with PTAA as HTL on breakdown voltages (*V*_RB_) exceeding ‐15 V, combined with the electrode design^[^ ^10^ ^]^ and high reverse bias lifetime (T_90_, ‐2.0 V, 100 h) through interface mobile iodides capture layer design^[^ ^12^ ^]^ demonstrates that the PTAA has a better application prospect in the preparation of high stability PSCs. However, the bottom‐buried HTL/perovskite interface has also been proven to be a dominant site for carrier loss in the p‐i‐n PSCs.^[^ ^13^ ^]^ There are easily formed high densities of nanovoids and deep‐level defects (e.g., Pb^2+^ dangling bond or Pb‐cluster),^[^ ^14^, ^15^ ^]^ leading to severe carrier non‐radiative recombination. Moreover, for PTAA HTL, a trade‐off between high conductivity and low coverage must be reached to improve its device performance and stability further.^[^ ^16^ ^]^ It led to perovskite films on PTAA exhibiting a low PLQY (<1%) due to severe nonradiative recombination loss at their interface.^[^ ^17^ ^]^ Consequently, rational interface tailoring to eliminate the bottom buried defects in perovskite and the pinhole in PTAA HTL is crucial for high‐performance and stable PSCs.

Generally, it is easy to have lots of excess lead halide at the top or buried bottom interfaces, which can vary the surface electronic states and behave like traps.^[^ ^5^, ^13^, ^18^ ^]^ In contrast to top surfaces, the bottom perovskite‐HTL interface is much more difficult to control. Attempts have been made to mix defect passivation species with HTLs,^[^ ^4^, ^5^, ^19^, ^20^ ^]^ given that many passivation molecules may be washed away during perovskite coating because of their high solubility in perovskite precursor solvents, such as 2‐methoxyethanol (2‐ME), dimethylformamide (DMF), and dimethyl sulfoxide (DMSO). In addition, new voids formed at the bottom interfaces along grain boundaries during long‐term device operation bring challenges for practical commodity applications. The weak interface between perovskites and the underlying ITO/PTAA substrates is demonstrated to be the key to the degradation of perovskite.^[^ ^21^ ^]^ Though modification PTAA via conductivity improvement,^[^ ^22^ ^]^ band tuning,^[^ ^23^, ^24^ ^]^ mechanical strengthening,^[^ ^25^ ^]^ hydrophilic ameliorating,^[^ ^26^ ^]^ and structural texture design^[^ ^27^ ^]^ have led to improved PCEs for PTAA‐based PSCs. However, to our knowledge, there is still a lack of studies that can simultaneously eliminate the nanovoid defects, anchor the excess lead halide at the buried bottom interface, and tailor the facet orientation of perovskite crystals without reducing the efficiency of hole extraction.

Herein, we report a method of constructing nanoscale localized lead‐chelating insulator bottom contacts to suppress carriers’ non‐radiative recombination and improve hole extraction at the PTAA/perovskite bottom interface. The well‐designed ultrathin nanoscale localized contact (abbreviated as UNLC) poly (methyl methacrylate) (PMMA) can passivate the excess Pb^2+^, fill the PTAA film's pinholes, improve the wettability of the PTAA film, and trigger the growth of perovskite films with the (111) facet to be the dominant orientation. This strategy enables high‐quality perovskite films with minimized defects at the buried interface. Thus, the resulting inverted PSCs achieved a PCE of 22.6% with a large improvement in open‐circuit voltage (*V*_OC_, 1.15 V) and fill factor (*FF*, 81.2%). Besides, the resulting PSCs retain 82% of the highest efficiency under ISOSL‐2 after MPP tracking for 982 h at 65 °C.

## 2 Results and Discussion

### 2.1 Defective Buried Interface and Its Healing by Insulating Polymer

**Figure 1:**
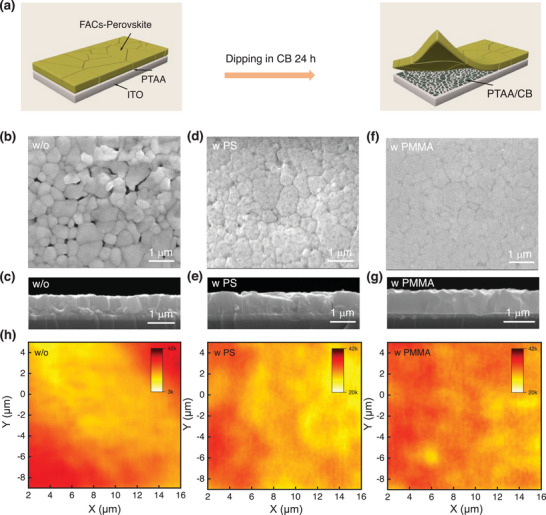
Morphology and optical characterization of the buried interface. a) Schematic of the lift‐off process: the bottom interface of the perovskite films is obtained by dipping the film in CB for over 24 h. b–g) The bottom morphology of the perovskite films with and without PMMA or PS, and the corresponding cross‐sectional SEM images. h) The PL‐mapping of the perovskite buried bottom interface without and with the UNLC of PMMA or PS on PTAA.

The PTAA film thickness is the key factor that dominates the PTAA‐based PSCs’ performance. High‐thickness PTAA film can bring high reverse bias stability,^[^ ^10^ ^]^ but it also poses a large challenge to high‐performance PSCs (e.g., low conductivity or doping‐induced low stability).^[^ ^28^, ^29^, ^30^ ^]^ To this end, an ultra‐thin layer of PMMA or polystyrene (PS) is well‐designed to achieve the trade‐off between high conductivity and low coverage for PTAA HTLs. As shown in **Figure** **1**a, the buried bottom interface is exposed by peeling the FA_0.90_Cs_0.10_PbI_2.83_Br_0.17_‐based perovskite film from the ITO‐glass substrate after dipping the film into chlorobenzene (CB) solvent for over 24 h. The buried bottom interface of the perovskite film is proven to have many nanocavity defects and/or disorder grains from the scanning electron microscopy (SEM) images and cross‐sectional SEM images when the PTAA is used as the bottom crystal growth interface (Figure 1b,c). Notably, the buried nanocavity defects are difficult to cure by optimizing the PTAA film thickness (Figure S1, Supporting Information). The reason for this problem may originate from the poor wettability and/or the residual strain during the film thermal annealing process.^[^ ^31^ ^]^ As shown in Figure S2 (Supporting Information), the wettability of the PTAA film can be adjusted a bit by reducing the thickness of the PTAA film, along with the reduction of the nanocavity defects. However, it cannot inhabit these buried defects only by adjusting the thickness of the PTAA film. Moreover, the decrease in PTAA film thickness also brings a risk of larger leak current and more carrier non‐radiation loss due to the lower coverage and many more pinholes.^[^ ^16^ ^]^ It leads to the device with poor stability (e. g. poor reverse bias stability) and low performance. Notably, the nanovoids on the bottom surface are much reduced when PS or PMMA is well‐designed to cover PTAA films (Figure 1d,f). Similarly, this phenomenon is found in the top SEM images (Figure S3, Supporting Information). However, there are still some large cracks at the grain boundaries for the PS sample from the cross‐sectional SEM image (Figure 1e). More vertically oriented and dense grains can be distinguished in the PMMA sample (Figure 1g). The PL intensity of the buried bottom surface of the PMMA sample is uniform and significantly higher than that of the control and PS samples, indicating the much‐reduced non‐radiative recombination loss of charge carriers (Figure 1h). In addition, the well‐designed PMMA or PS can enhance the wettability of the PTAA film for perovskite precursor solution and reduce the root‐mean‐square (RMS) roughness of PTAA films (6.15 to 1.95 nm, PMMA modification), which is beneficial for constructing perovskite films with a higher crystallinity and fewer non‐radiative recombination sites (Figures S4 and S5, Supporting Information).

We observed the crystallization process of perovskite precursor solutions on different substrates over time, finding that the crystallization rate of precursor solutions on the PTAA/PMMA substrate is the fastest, followed by PS, and then the bare PTAA substrate. It may be ascribed to the highest wettability of the PTAA/PMMA substrate and the chelating effect between PMMA and PbI_2_ (Figure S6, Supporting Information). Thus, the PMMA has a stronger modification effect than PS in inhibiting bottom defects and regulating the crystallization process of the perovskite film.

### 2.2 The Mechanism and Effect of PMMA Modification

**Figure 2:**
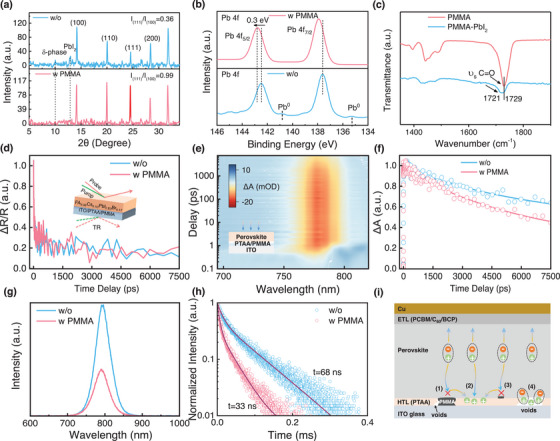
The effect mechanism of the UNLC of PMMA on PTAA. a) XRD pattern, b) Pb 4f XPS spectra of the perovskite films without and with PMMA measured from the bottom surface (ITO/PTAA, ITO/PTAA/PMMA). c) FTIR spectra of PMMA and PbI_2_‐PMMA. d) The transient reflectance (TR) spectroscopy of the perovskites from the bottom surface. e) Pseudo‐color TA plots, where a 400 nm pump pulse generated by an fs‐laser was used to excite the TA signal. f) Dynamic TA decay of perovskite phase probed at GSB peak of 786 nm, g) Steady‐state photoluminescence (PL) spectra, h) Time‐resolved PL (TRPL) spectra for films without and with PMMA‐modification. i) The schematic diagram for the effect mechanism of the ultrathin nanoscale local contact of PMMA.

More detailed studies are conducted to understand the effect mechanism for PMMA modification. As shown in **Figure** **2**a, the bottom surface XRD spectra features much resident lead iodide and the non‐perovskite phase (i. e. δ‐phase) for the control samples, which is much reduced for the PMMA‐modified sample. The result indicates that the modified bottom PTAA interface by PMMA regulated the crystallization process of the perovskite film and improved its crystal quality. This result is consistent with what was previously reported, where PMMA can serve as a polymer template to control nucleation and crystal growth.^[^ ^32^ ^]^ Moreover, the PMMA‐modified perovskite film exhibits a higher I_(111)_/ I_(100)_ intensity ratio of 0.99 in comparison with that of 0.36 for the control film, suggesting that the local PMMA can serve as a template to finely modulate the perovskite film facet orientation to be (111)‐dominated. The reason may be that the interaction between PMMA molecules and the (111) crystallographic plane is the most robust.^[^ ^33^, ^34^ ^]^ The (111) facet is not vulnerable to cationic migration than the (100) facet,^[^ ^4^, ^19^ ^]^ indicating it can improve the device stability. As shown in Figure S7 (Supporting Information), the top surface XRD spectra also show this phenomenon. The high‐resolution X‐ray photoelectron spectroscopy (XPS) spectra of Pb and I at the bottom surface for these two perovskite films are shown in Figure 2b and Figure S8 (Supporting Information). Compared to the control film, the main peaks of Pb 4 f (142.45 eV for Pb 4f_5/2_ and 137.65 eV for Pb 4f_7/2_) shifted toward higher binding energy regions (142.82 eV for Pb 4f_5/2_ and 137.95 eV for Pb 4f_7/2_) in PMMA‐modified perovskite film due to the strong coordination between C═O groups in PMMA and the under‐coordinated Pb^2+^ defect.^[^ ^5^ ^]^ There are two small peaks located at 135.28 and 140.18 eV due to the presence of metallic lead species (Pb^0^), which have served as non‐radiative recombination centers to impair solar cell operation. Notably, the presence of a substantial amount of atomic Pb indicates the existence of iodide vacancies in the control film. In our recent study, the iodide species defect can severely impact the device's performance and operational stability.^[^ ^12^, ^20^ ^]^ Thus, a lower level of Pb defects is one of the key factors in improving the long‐term stability of the device. The coordination between C═O groups in PMMA and PbI_2_ is also supported by Fourier transform infrared spectroscopy (FTIR) spectra. As shown in Figure 2c, the shift of the C═O vibration in PMMA to a lower wavenumber upon interaction with PbI_2_ indicates the formation of an intermediate PMMA‐PbI_2_ adduct, which can regulate the crystal growth and improve the crystallinity of the perovskite film. In addition, the enhanced interface bonding intensity (Figure S9, Supporting Information) between perovskite and PTAA indicates that PMMA has formed stable chemical bonds at the interface between PTAA and perovskite.^[^ ^35^, ^36^ ^]^

The charge carrier transport capability of the PMMA modified PTAA HTL is evaluated using transient reflection (TR) spectroscopy. Figure 2d and Figure S10 (Supporting Information) show the TR spectra of perovskite films without and with PMMA modification. The films are excited from the bottom using a 400 nm pump laser with a penetration depth of less than 30 nm.^[^ ^37^ ^]^ The nearly identical charge carrier lifetimes at the PTAA and PTAA‐PMMA interfaces indicate that the UNLC of PMMA does not hinder carrier transport or collection processes. Moreover, the current‐voltage (*I–V*) characteristics of the ITO/PTAA/Au and ITO/PTAA/PMMA/Au further show that the appropriate PMMA modification does not significantly affect the conductivity of the PTAA film (Figure S11, Supporting Information).

To further analyze the impact of PMMA modification, the transient absorption (TA) spectra are measured for both films (Figure 2e; Figure S12, Supporting Information). Carrier dynamics are extracted by monitoring the ground‐state bleaching (GSB) peak at 786 nm (Figure 2f). The PMMA‐modified sample exhibited a faster charge transfer rate compared to the control sample, demonstrating the positive impact of PMMA modification on carrier transport efficiency. Additionally, time‐resolved photoluminescence (TRPL) and steady‐state photoluminescence spectra (Figure 2g,h; Table S2, Supporting Information) further confirm the significantly enhanced crystallinity of the PMMA‐modified perovskite film. The UV–vis absorption spectra (Figure S13, Supporting Information) show that the introduction of PMMA does not alter the bandgap of the perovskite film. The enhancement of absorbance is ascribed to the improved film quality and increased film thickness (Figure S14, Supporting Information). These results collectively highlight that PMMA modification improves film quality and charge carrier dynamics without compromising optical or electronic properties.

The schematic diagram for the effect mechanism of the UNLC of PMMA is shown in Figure 2i. The introduced localized contact PMMA may have multiple functions: 1) Filling the pinholes without changing the original energy level structure of PTAA. 2) Formation dipole at the PTAA/perovskite interface to increase the built‐in potential in the PSCs.^[^ ^38^ ^]^ 3) Supporting a template to promote the crystal growth with a specific facet by coordinating with PbI_2_. 4) Passivating the uncoordinated Pb^2+^ and reducing the iodine species defects. Meanwhile, the minimal reduction of the area of the charge transport layer (i. e. PTAA) will not damage its photogenerated carriers extraction ability, for the carriers can bypass the insulation layer to reach the HTL region (paths 1–3 in Figure 2i) or tunneling into the HTL.^[^ ^39^, ^40^ ^]^ Generally, the photocarrier will be recombined at defective contact sites, such as path 4 in Figure 2i. (The corresponding photocarrier transport pathways are illustrated in the region of 1, 2, 3, 4 in Figure 2i), the reduction of the contact area will suppress the non‐radiative recombination of carriers.^[^ ^41^, ^42^ ^]^

**Figure 3:**
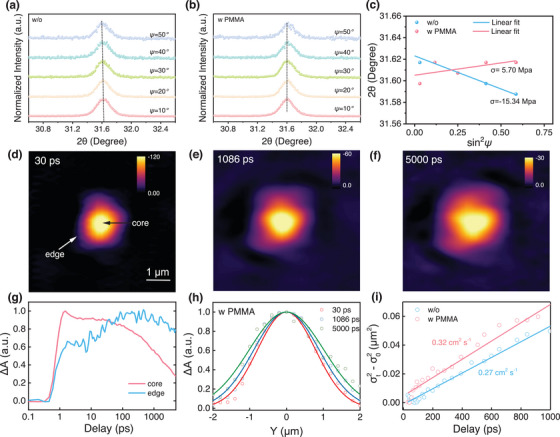
Strain analysis and in situ study of carrier diffusion dynamics. GIXRD patterns at different instrument tilt angles for samples a) without and b) with PMMA, c) Linear fit of 2*θ*‐sin^2^ *ψ* of these two samples. d–f) The transient absorption microscopy (TAM) images of carrier transport dynamics of the PMMA‐modified perovskite with delay times of 30, 1086, and 5000 ps, respectively. The images illustrate the outward diffusion of excitons from the excitation site over time. g) Temporal evolution of transient absorption signal intensity at the core and edge, indicating carrier transport. h) Gaussian fits of excited‐state density profiles along the *Y*‐axis for the PMMA‐modified films at different delay times. i) Diffusion coefficients of perovskite films without and with PMMA.

The residual stress state is checked using the depth‐resolved grazing incident X‐ray diffraction (GIXRD) technique with the 2*θ*‐sin^2^ *ψ* method to investigate the effect of UNLC of PMMA on the film crystallinity.^[^ ^43^, ^44^ ^]^ As displayed in **Figure** **3**a, for the control sample, the characteristic (012) crystal plane diffraction peaks show an obvious shift to lower angles by varying the angle *ψ* of the instrument from 10° to 50°, indicating the gradually increased crystal plane distance according to Bragg's Law. The negative slope of the linear fitting of 2*θ*‐sin^2^ *ψ* demonstrates tension stress in the control sample. According to Bragg's law, Hooke's law, and equilibrium equations, the residual stress (i.e., *σ*) can be obtained as follows.

(1) $$\sigma =-\frac{E}{2\left(1+\nu \right)}\frac{\pi }{180^{\circ }}\cot\theta_{0}\frac{\partial \left(2\theta \right)}{\partial \sin^{2}\psi }$$

where *E* and *υ* are Young's modulus and Poisson's ratio of the thin film, respectively. *E* and *ν* are evaluated as 11.45 GPa and 0.3, respectively.^[^ ^45^ ^]^ *θ*_0_ is the diffraction peak of a stress‐free perovskite crystal plane, which may be obtained from the intersection of 2*θ* versus sin^2^ *ψ* lines for the same deposit in different stress states.^[^ ^46^ ^]^ Based on Equation (1), the residual strain in the control film is calculated to be ‐15.34 Mpa, whereas the PMMA‐modified perovskite film displayed a compressive strain of 5.70 Mpa (Figure 3c). This result demonstrates that the UNLC of PMMA can effectively regulate the tension strain to compressive strain in these films, which helps to improve device photovoltaic performance and long‐term stability. A schematic diagram of the strain regulation for control and PMMA samples is illustrated in Figure S15 (Supporting Information).

The effect of UNLC of PMMA on charge carrier transport dynamics is investigated further by transient absorption microscopy (TAM). TAM supports the study of the spatial and temporal evolution of carrier diffusion, which makes up for the lack of spatial resolution information about carrier dynamics in the TA test. In the TAM measurements, a 650 nm femtosecond pump pulse is focused on the film surface to photoexcite electrons and holes. The spatial diffusion of these carriers is monitored over time for both PMMA‐modified and control films (Figure 3d,f; Figure S16, Supporting Information). The excitons generated at the excitation site diffused outward, forming Gaussian intensity distributions as a function of delay time. The temporal evolution of the signal along the *Y*‐axis was fitted with Gaussian functions, revealing an increase in the full width at half maximum (FWHM) over time (Figure 3g,h; Figure S17, Supporting Information). This behavior reflects the outward diffusion of carriers from the initial excitation region. The diffusion coefficient is determined by fitting the variance of the Gaussian profiles to the equation $\sigma_{x}^{2}\left(t\right)=\sigma_{0,x}^{2}\left(t\right)+2Dt$.^[^ ^47^ ^]^ The PMMA‐modified films exhibited a higher diffusion coefficient, increasing from 0.27 cm^2^ s^−1^ (control) to 0.32 cm^2^ s^−1^ (w PMMA). This improvement is attributed to the high crystalline quality and the optimized facet orientation of the PMMA‐modified perovskite. These results also suggest that UNLC of PMMA can regulate the buried bottom interface and bulk film crystallinity simultaneously.

### 2.3 Photovoltaic Performances and Stability of the PSCs after PMMA Modification

**Figure 4:**
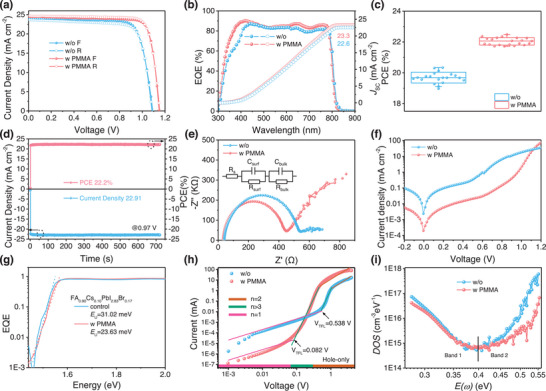
The performance and electrical characterization of the PSCs. a) Current density–voltage (*J–V*) curves for the champion control and PMMA PSCs measured under reverse scan (RS) and forward scan (FS). b) External quantum efficiency (EQE) spectra and the corresponding integrated *J*_SC_. Statics of c) PCEs for over 20 devices without or with PMMA modification. In a typical box plot, the line in the box is the median line, and the square in the center of the box is the average point. d) Stabilized current density and PCE in FA_0.90_Cs_0.10_PbI_2.83_Br_0.17_ based PSCs with PMMA. e) Nyquist plots of EIS with the equivalent circuit, f) Dark current‐voltage curves, and g) *E*_U_ extracted by band edge fitting of the exponentially plotted EQE spectra for the devices with or without PMMA. (h) Dark current‐voltage curves of hole‐only devices with or without PMMA. i)Trap density of states (Tdos) for PSCs with or without PMMA.

The FA_0.90_Cs_0.10_PbI_2.83_Br_0.17_ PSCs with a device structure of ITO/PTAA (PTAA/PMMA)/ Perovskite/PCBM/C_60_/BCP/Cu are prepared to confirm the PMMA modification. Compared with the untreated devices (**Figure** **4**a; Figure S18 and Table S2, Supporting Information), the open‐circuit voltage (*V*_OC_) and the Fill Factor (*FF*) in PTAA/PMMA‐based PSCs increased from 1.09 to 1.15 V and from 76.7% to 81.2%, respectively. These improvements illustrate a higher carrier collection rate and a lower carrier non‐radiative recombination loss, leading to an increased PCE from 20.3% to 22.6%. Moreover, the PMMA‐modified PSCs show higher external quantum efficiency (EQE) in the 320–460 nm and 600–800 nm regions. It is ascribe to the reduced buried defect density and the increased thickness of the perovskite film,^[^ ^18^ ^]^ resulting in a high integrated *J*_SC_ (23.3 mA cm^−2^) in agreement with that from *J–V* curves (Figure 4b; Figure S14, Supporting Information). The statistical distribution of PCEs for over 20 PSCs without and with PMMA in Figure 4c demonstrates much improved efficiency and better device reproducibility after PMMA modification. The stabilized power output of FA_0.90_Cs_0.10_PbI_2.83_Br_0.17_ PSCs under AM1.5G illumination is tested at the maximum power point (MPP) with a fixed voltage of 0.97 V. The PCE stabilized at 22.2%, indicating the reliability of high device performance (Figure 4d).

The electronic impedance spectroscopy (EIS) characterizations shown in Figure 4e demonstrate that the UNLC of PMMA does not improve the series resistance (*R*_S_, 33.2 Ω to 41.1 Ω for the devices with or without PMMA, respectively) of the interface between PTAA and perovskite, which plays a key role in obtaining a high *FF* in the PSCs.^[^ ^48^, ^49^ ^]^ The reduced *R*_S_ and a much higher recombination resistance (*R*_rec_) after PMMA modification guarantee the high *FF* and *V*_OC_ of PSCs (Figure S19, Supporting Information). In addition, the dark‐current test shows the lower reverse saturation current and higher rectification ratio of the PSCs fabricated with the PTAA/PMMA relative to PTAA, suggesting the reduced leakage current (Figure 4f), which is consistent with the result in Figure 1b–g.

In principle, low‐energy disorder films with reduced band tail states exhibit a minimum of the *V*_OC_ deficit. To evaluate the energy disorder in these films, we extracted the Urbach tail (*E*_U_).^[^ ^50^ ^]^ Figure 4g shows that the perovskite with PMMA exhibits much lower *E*_U_ (23.63 meV) than the control perovskite (31.02 meV). The defect chemistry for PMMA‐modified PSCs is further quantitatively studied. As shown in Figure 4h and Table S3 (Supporting Information), the PMMA‐modified devices show lower hole trap densities (5.29 × 10^14^ cm^−3^) than the untreated ones (3.47 × 10^15^ cm^−3^). The hole mobility for the PMMA‐modified sample increased by about one order of magnitude (7.42 × 10 ^−4^ to 6.01 × 10 ^−3^ cm^−3^ V^−1^ s^−1^). In addition, the thermal admittance spectroscope (TAS) demonstrates that the PMMA‐modified device had lower tDOS in the deeper trap region (band II, > 0.40 eV), which is assigned to the dominant iodide interstitials (I_i_^−^ and I_i_^+^) defects at the film surface (i.e., top and/or bottom surface).^[^ ^51^, ^52^ ^]^ This indicates that the UNLC of PMMA exhibits better defect management and crystallization regulation function.

**Figure 5:**
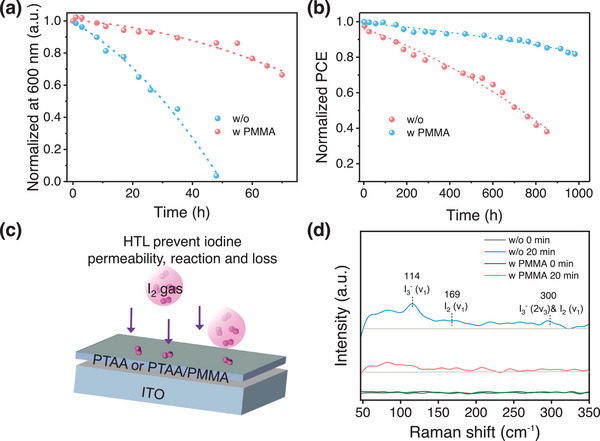
Stability of the FA_0.90_Cs_0.10_PbI_2.83_Br_0.17_‐based PSCs. a) Photo‐oxygen stability of the absorption in perovskite films (normalized at 600 nm) under a combination of pure oxygen (10^5^ Pa) and white LED (1 sun) treatment. b) Operational stability during MPPs tracking of PSCs under 1 sun continuous illumination (LED lamp) and 65 °C aging. c) Schematic diagram of the transport and interaction between I_2_ and HTL. d) Raman spectra of the indicated PTAA HTLs without and with PMMA before and after I_2_ exposure for 20 min.

The influence of the UNLC of PMMA on the PSCs' operational stability is investigated. The photo‐oxygen stability of FA_0.90_Cs_0.10_PbI_2.83_Br_0.17_ PSCs is studied to confirm the reduction of iodide species defect density by the coordination effect between PMMA and uncoordinated Pb^2+^, and the suppressed ion migration by the template‐induced (111) facet orientation growth. The iodine vacancy (V_I_) concentration has proven to be directly related to the photo‐oxidative stability due to its similar ionic radius to oxygen molecules.^[^ ^48^ ^]^ As shown in **Figures** **5**a and S15 (Supporting Information), the FA_0.90_Cs_0.10_PbI_2.83_Br_0.17_ film shows sharply decreased absorption (at a wavelength of 600 nm) after 11 h (less than 80% of initial absorption at 600 nm) and decomposes to a PbI_2_ phase dominant mixture after 35 h, indicating there are lots of defects in the film (i.e., V_I_). The PMMA‐modified sample demonstrates superior photo‐oxygen stability, with the absorption decrease rate reduced by over 5 times (T_80_, 11 h for control and 55 h for PMMA modification), which can be attributed to the fine‐manipulated iodine species defect density.

The normalized PCEs over time are depicted in Figure 5b, obtained under the condition of 1 sun continuous light illumination (1 sun, LED lamp) near maximum power points (MPPs). The MPPs are achieved by connecting the devices with a resistor (420 Ω) in series. Devices without PMMA show rapid decay after 232 h of aging (81% of initial PCE), possibly due to an abundance of bottom‐buried cavities and plenty of uncoordinated ion defects on the film. In contrast, PMMA‐modified PSCs retain 82% (90%) of initial PCE after 982 (635) h of operation, meeting the requirements of the ISOS‐L‐2 protocol.^[^ ^53^ ^]^

The mechanism by which PMMA enhances the stability of the device is studied further. During the operation of the device, the newly generated iodine species defects have a significant impact on the device's operational stability.^[^ ^12^, ^54^ ^]^ Figure 5c is the schematic diagram of the transport and interaction between I_2_ and HTL. Figure 5d shows the Raman spectroscopy measurements on these HTLs before and after I_2_ exposure. The electrons in the HOMO of the HTL can transfer to I_2_, forming [HTL]^+^/I_3_^−^ or [HTL]^+^/I^−^ compounds and trapping iodine species in the HTL.^[^ ^55^ ^]^ The significant decrease in Raman shift peaks for iodine species (i.e., I^−^, I_2_, I_3_^‐,^ etc) for PTAA with PMMA film may be related to the fact that PMMA with a deep HOMO level can cut off the redox reaction between HTL and I_2_ (Figure S16, Supporting Information).^[^ ^56^ ^]^ Meanwhile, the PMMA can also physically block the penetration of I_2_ into PTAA. It effectively blocks the formation of I_3_^−^ and I^−^ species and slows I_2_ permeation in the HTL.^[^ ^55^, ^57^ ^]^ This is beneficial for improving the working stability of the PSCs. During the operation of the device, the iodine species generated in the film penetrate the PTAA and react with it, providing an extra iodine transport pathway, which will affect the performance and stability of the device.^[^ ^55^ ^]^

## 3 Conclusion

In this work, an ultrathin nanoscale localized contact insulating polymer is introduced to the PTAA/perovskite interface for synergistic modification of both sides. The localized contact insulating polymer layer PMMA can both act as an HTL dressing agent and the buried uncoordinated Pb^2+^ chelating agent to form a well‐designed interface with much reduced non‐radiative recombination sites and leak current paths. The PMMA can fill the pin holes in the PTAA HTL film and acts as a crystal growth template to promote the growth of perovskite crystals with (111) as the dominant crystal facet. It reduces the iodine species defect density and suppresses ion migration in the perovskite film, and prevents the permeation of I_2_ to the PTAA HTL. Finally, a performance of 22.6% and much‐enhanced stability (e.g., stability of T_80_ for photo‐oxygen increased over 5 times, light‐thermal over 4 times) of FA_0.90_Cs_0.10_PbI_2.83_Br_0.17_‐based PSCs is demonstrated. This study provides new insight into the well‐designed insulating polymer passivation for achieving low‐defect, high‐quality, and well‐oriented facets for perovskite films, providing more opportunities for promoting PSCs’ commercialization.

## Conflict of Interest

The authors declare no conflict of interest.

## Supporting information



Supporting Information

## Data Availability

The data that support the findings of this study are available from the corresponding author upon reasonable request.
