# Supplementary material for: Localized Lead‐Chelating Insulator Bottom Contact for Efficient and Stable p‐i‐n Perovskite Solar Cells
Source: Adv Sci (Weinh). 2025 Jul 18;12(39):e09816. doi: 10.1002/advs.202509816 (PMC12533327; doi:10.1002/advs.202509816)
Supplement: Supplementary file 1 — Supporting Information [file ADVS-12-e09816-s001.pdf]

ADVANCED SCIENCE
Open Access

Supporting Information

for *Adv. Sci.*, DOI 10.1002/advs.202509816

Localized Lead-Chelating Insulator Bottom Contact for Efficient and Stable p-i-n Perovskite Solar Cells

Ying Li, Yan Chen, Kang Ding, Yuanyuan Guo, Yuhan Liu, Shiwei Lu, QingQing Zou, Rongzhou Liang, Sijin Liu, Zhimin Liu, Haipeng Xie, Dongsheng Tang, Le-Man Kuang, Yaxin Zhai and Jifei Wang**

Supporting Information

Localized Lead-chelating Insulator Bottom Contact for Efficient and Stable p-i-n Perovskite Solar Cells

Ying Li†, Yan Chen†, Kang Ding, Yuanyuan Guo, Yuhan Liu, Shiwei Lu, QingQing Zou, Rongzhou Liang, Sijin Liu, Zhimin Liu, Haipeng Xie, Dongsheng Tang, Le-Man Kuang, Yaxin Zhai and Jifei Wang**

Y. Li, Y. Chen, K. Ding, Y. Liu, S. Lu, Q. Zou, R. Liang, S. Liu, D. Tang, L-M. Kuang, Y. Zhai, and J. Wang

Key Laboratory of Low-Dimensional Quantum Structures and Quantum Control of Ministry of Education, Institute of Interdisciplinary Studies, Department of Physics, Hunan Normal University, Changsha 410081, China.

*E-mail： wangjf@hunnu.edu.cn, yzhai@hunnu.edu.cn

Y. Li, Y. Chen, K. Ding, Y. Liu, S. Lu, Q. Zou, R. Liang, S. Liu, D. Tang, L-M. Kuang, Y. Zhai, and J. Wang

Hunan Research Center of the Basic Discipline for Quantum Effects and Quantum Technologies, Hunan Normal University, Changsha 410081, China.

Y. Li, Y. Chen, K. Ding, Y. Liu, S. Lu, Q. Zou, R. Liang, S. Liu, D. Tang, L-M. Kuang, Y. Zhai, and J. Wang

Key Laboratory of Multifunctional Ionic Electronic Materials and Devices, School of Physics and Electronics, Hunan Normal University, Changsha 410081, China.

Y. Guo

Institute of Flexible Electronics (IFE), Northwestern Polytechnical University, Xi'an, 710072, Shanxi, China.

Z. Liu

School of Science, East China Jiaotong University, Nanchang 330013, China.

H. Xie

Hunan Key Laboratory of Super Microstructure and Ultrafast Process, School of Physics and Electronics, Central South University, Changsha, Hunan 410083, China.

† These authors contributed equally to this work.

Experimental Section

Materials

N, N-dimethylformamide (DMF, 99.8%, anhydrous), dimethylsulfoxide (DMSO, 99.8%, anhydrous), chlorobenzene (CB, 99.8%), IPA (anhydrous, 99.8%), PbI_2 (99.999%), PbBr2 (99.999%), CsI (99.99%), polystyrene (PS, 10%(solids)), and polymethyl methacrylate (PMMA, Powder (48 micron)), Bathocuproine (BCP, 99.99%) were purchased from Sigma-Aldrich. Polymethyl Methacrylate (PTAA, 99.8%) was purchased from Xi'an Polymer Light Technology Corp. [6,6]-phenyl-C61-butyric acid methyl ester (PCBM) was purchased from Luminescence Technology Corp. Fullerene (C60, 99.95%) was purchased from Nano-C. Formamidinium iodide (FAI, 99.9%) was purchased from Great Cell Energy.

Solution preparation

$FA_{0.90}Cs_{0.10}PbI_{2.83}Br_{0.17}$ perovskite. FAI (2.91 M), $PbBr_2$ (1.36 M) and PbI_2 (1.41 M) were dissolved in DMF: DMSO = 9:1 (v/v) mixed solvent, and CsI (1.50 M) was dissolved in DMSO. The 'mixed' perovskite precursor solutions of $FA_{0.90}Cs_{0.10}PbI_{2.83}Br_{0.17}$ were prepared with FAI (0.90 M), PbI_2 (0.92 M), $PbBr_2$ (0.085 M) and CsI (0.10 M) from the predissolved solution; this perovskite precursor solution was then aged for 6-8 h at 65 °C before use.

Device fabrication

The ITO glass was cleaned with detergent, deionized water, acetone, ethanol, and IPA successively. PTAA was dissolved in chlorobenzene to form 2.0 mg mL^{-1} solution and then spin-coated on pre-cleaned indium tin oxide (ITO) substrate at 6000 rpm for 30 s and annealed at 65 °C for 10 mins in N_2.

For $FA_{0.90}Cs_{0.10}PbI_{2.83}Br_{0.17}$ perovskite film, a one-step method was used. The perovskite precursor solutions of $FA_{0.90}Cs_{0.10}PbI_{2.83}Br_{0.17}$ was spin-coated at 6000 rpm for 30 s with pouring chlorobenzene as an anti-solvent. Then the substrates were annealed at 100 °C for 20 min in N_2. PCBM with a concentration of 15 mg mL^{-1} in CB was spin coated on perovskites at 3000 rpm for 30 s and annealed at 80 °C for 10 mins.

As for PTAA films coated by PS or PMMA, the ultrathin nanoscale localized contact of PS (0.15 mg/ml in CB), or PMMA (0.09 mg/ml in CB) were fabricated by spin-coating at 4000 rpm for 30 s, respectively. Then it was annealed for 5 min at 65 °C on a hot plate.

At last, the C_{60} (10 nm thick), BCP (7 nm thick) and Cu electrode (70 nm thick) were thermally evaporated sequentially on the films in vacuum at a rate of 0.1 Å s^{-1}, 0.2 Å s^{-1}, and 2 Å s^{-1}, respectively. The device area is defined to be 0.092 cm^2 by metal masks.

Device testing

The current density (*J*)-voltage (*V*) curves of perovskite solar cells (PSCs) were measured in nitrogen glovebox by Keithley 2400 with a voltage scan rate of 0.02 V s^{-1}, delay time of 50 ms and sweep region from -0.2 V to 1.2 V under 100 mW cm^{-2} AM 1.5G illumination provided by an AAA-class solar simulator (Enli Technology Co., Ltd.). A NREL certificated Si reference cell (SRC-2020, Enli Technology Co., Ltd) was used for calibration. The external quantum efficiency (EQE) was characterized by the QE-R solar cell quantum efficiency measurement system (Enli Technology Co., Ltd.), and the light source is a 75 W xenon lamp. The monochromatic light intensity for EQE was calibrated with a NIST-certified Si photodiode from 300 nm to 1100 nm. The EQE spectrum was integrated over AM1.5G photon flux to attain photocurrent density.

Device operational stability test

Long-term operational stability measurements of unencapsulated perovskite devices were operated under a solar simulator (Newport 94011A) with a light intensity of 100 mW cm^{-2} AM1.5G in N_2. All devices were loaded with a resistor so that they worked at conditions near the MPP during the test. The light and thermal stability of unencapsulated PSCs were measured under a 1 sun condition (white LED lamp; the light intensity was adjusted to maintain fresh PSCs with a J_{SC} of 23 mA cm^{-2}) combined with 65 °C heating in N_2.

Film Characterization

The absorbance spectra were obtained by using a UV-Visible Spectrometer in the spectral range of 300-1100 nm. Steady-state Photoluminescence (PL) was measured by i-HR320 spectrometer

(HORIBA Scientific) with excitation by a UV laser (405 nm). The PL emission peak wavelength mapping images were calculated from the PL intensity mapping images at different emission wavelengths from 760 nm to 820 nm. The chemical state a of the samples was analyzed with XPS equipped with a monochromatic SPECS XR-MF X-ray source (Al Kα, $h\upsilon$ = 1486.7 eV) in ESCAKAB 250Xi system (base pressure: 2.0×10^{-10} mbar). X-ray diffraction (XRD) measurements were carried out with a Siemens D500 Bruke X-ray diffractometer (Cu Kα radiation, λ = 1.5406 Å). Raman scattering measurements on perovskite films were taken with an high resolution confocal Raman spectrometer (Horiba JY LabRAM HR Evolution) using a continuous wave laser with wavelengths of 532 nm as the excitation light source. Scanning electron microscopy (SEM) characterizations were performed by using a scanning electron microscope (TESCAN MIRA3 LMU) at an acceleration voltage of 20 KeV. The PL lifetime decay profile was recorded by the time-correlated single photon counting system (Picoquant "Timeharp 300"). The picosecond 5 pulsed laser diode with a wavelength of 375 nm (Picoquant "PDL 800-B", 10-80 MHz) was used as the excitation source.

Transient Absorption (TA) / reflection (TR) Spectroscopy: The TA measurements were carried out using a pump-probe spectrometer. A Ti: sapphire amplifier generates the fundamental laser pulse with a wavelength of 800 nm and a pulse repetition rate of 1 kHz. This fundamental pulse is divided into two parts by a beam splitter. A BBO crystal was employed for the second harmonic generation of the 800 nm wavelength to generate an excitation pump at 400 nm. The average excitation density is determined by dividing the input photon flux by the pump penetration depth. The other part of the fundamental pulse is focused into a sapphire crystal to create a white light continuum (450-850 nm) that serves as the probe. A motorized translation stage with a retroreflecting mirror is used to delay the probe pulses in time relative to the pump pulses. The pump and probe beam incident the sample normally and spatially overlap at the sample surface. The focused spot size for the probe and pump beams at the sample position is approximately 200 μm and 600 μm, respectively. For the TR measurements, the incident angle for both pump and probe is around 45°.

Transient absorption microscope (TAM) was carried out on a commercial transient absorption microscope (Time-Tech Spectra). For the fundamental laser pulse with a wavelength of 800 nm, one part is directed to an optical parametric amplifier (OPA) to produce a 650 nm pump, which is then chopped at 500 Hz and attenuated using neutral-density filter wheels. Both pump and probe beams are combined using a long-pass filter and directed toward a microscope (Olympus, magnification of 50×, numerical aperture of 0.75, working distance of 3 mm). The pump and probe beams were spatially overlapped on the sample surface, both being incident on the sample normally. The focused spot size of the pump beam on the sample surface was approximately 1 μm. The transmitted probe beam of the sample was focused onto another 50× objective and collected through a charged metal-oxide semiconductor (CMOS) camera (BASLER-ac made in Germany).

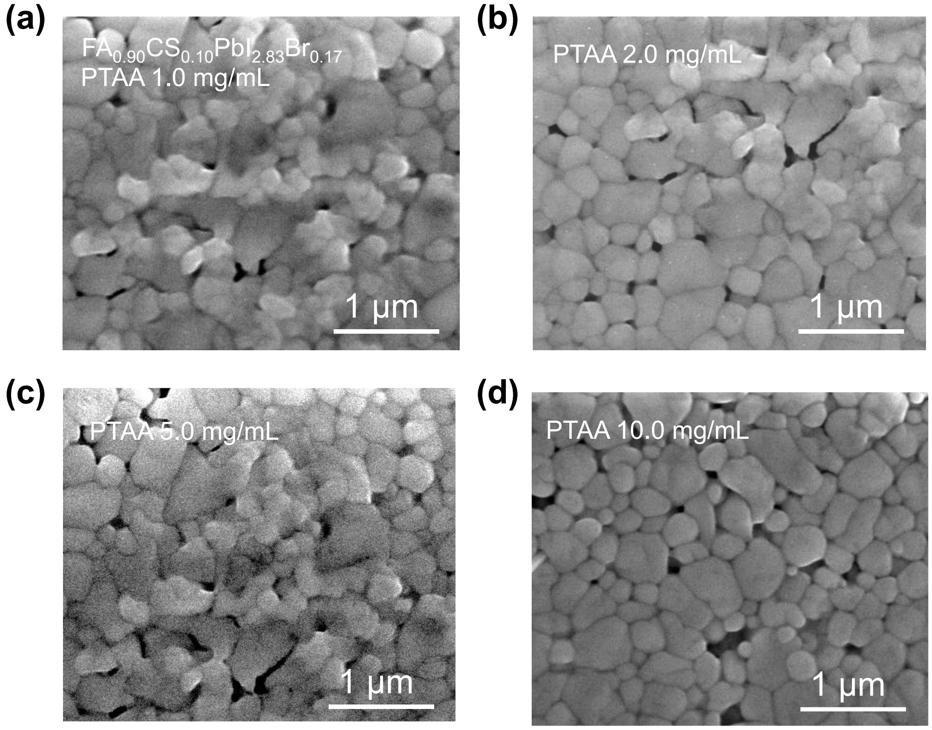

Figure S1. The scanning electron microscopy (SEM) images of the bottom buried surface of perovskite film deposited on PTAA with different concentrations (1.0 mg/ml; 2.0 mg/ml; 5.0 mg/ml; 10.0 mg/ml in CB).

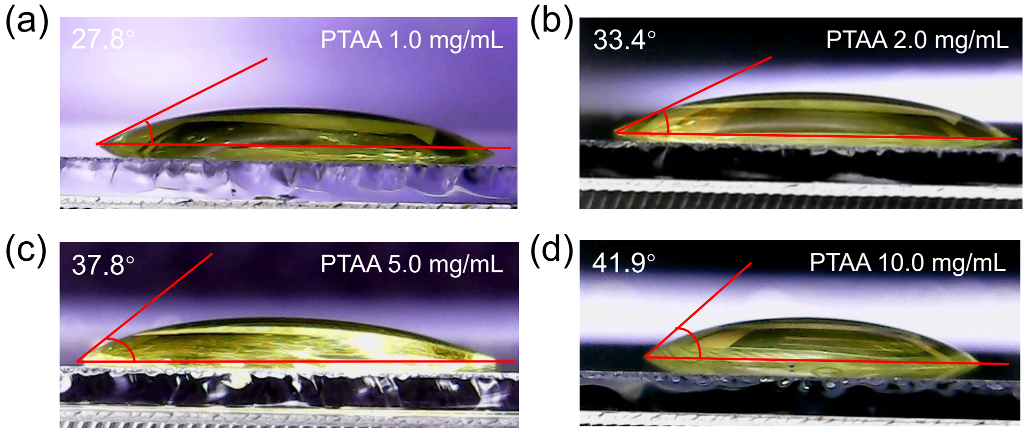

Figure S2. The contact angles of the perovskite precursor on PTAA with different PTAA concentrations (e.g. 1.0 mg/ml; 2.0 mg/ml; 5.0 mg/ml; 10.0 mg/ml in CB).

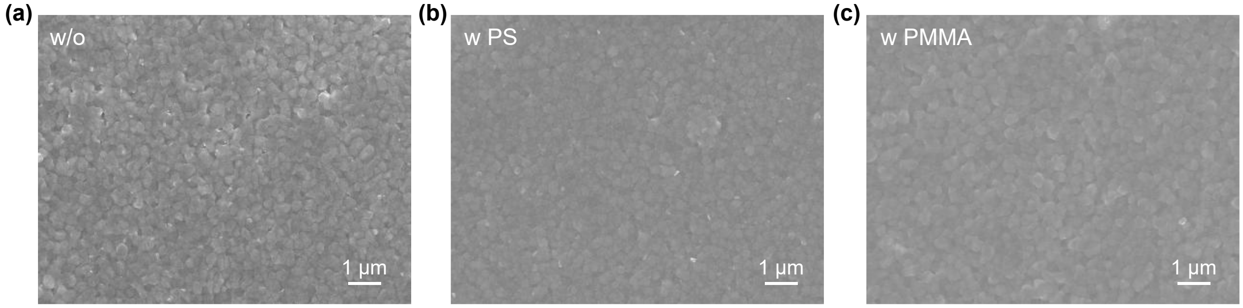

Figure S3. The top scanning electron microscopy (SEM) images of the perovskite films on PTAA (a) without or (b) with PS, (c) w PMMA, respectively.

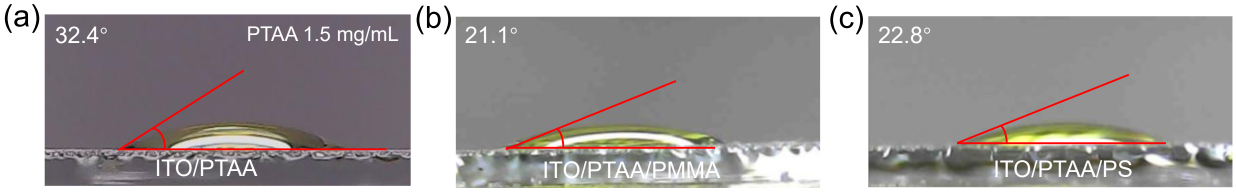

Figure S4. The contact angles of the hole transport films and the corresponding perovskite precursor solution: the as-deposited (a) ITO/PTAA, (b) ITO/PTAA/PMMA, (c) ITO/PTAA/PS.

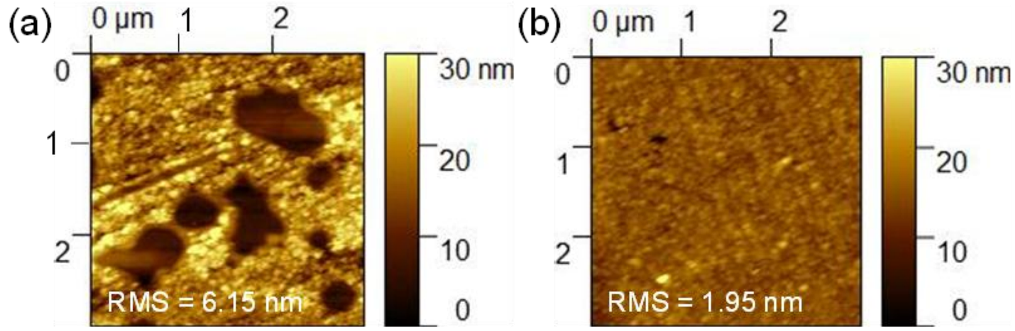

Figure S5. Atomic force microscopy (AFM) images of the PTAA films without (a) or (b) with PMMA-modification.

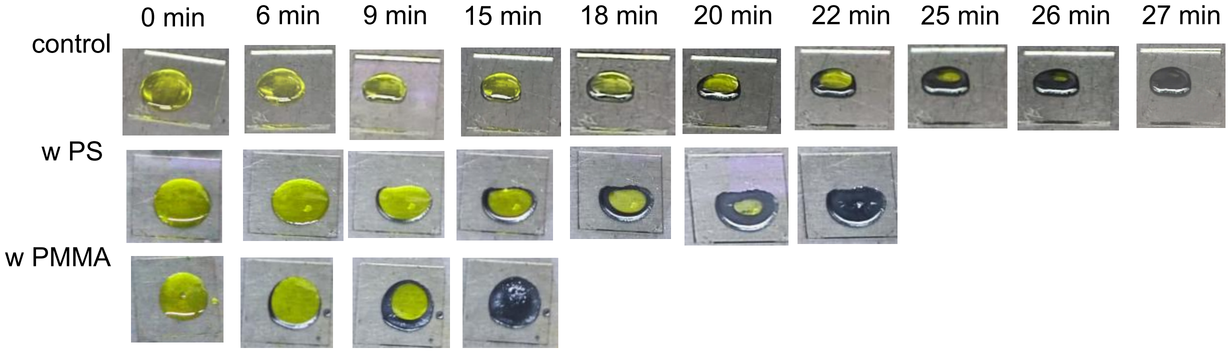

Figure S6. The crystallization of perovskite precursor solutions at different time for substrates of (a) ITO/PTAA, (b) ITO/PTAA/PS and (c) ITO/PTAA/PMMA.

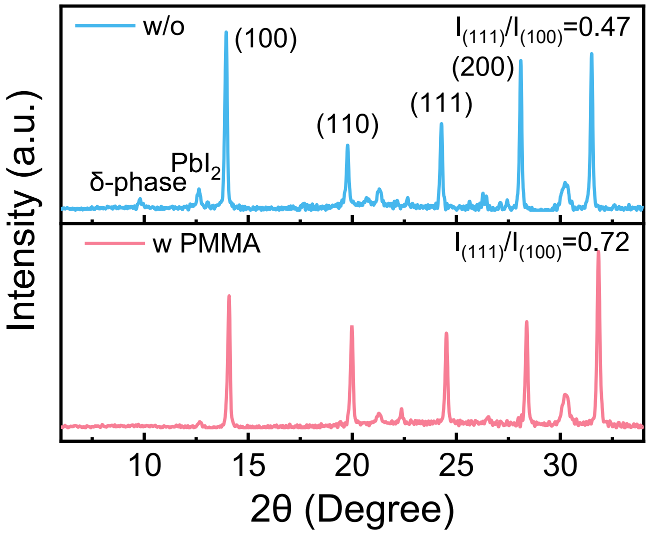

Figure S7. The top surface XRD spectra for the control and PMMA-modified perovskite films. The PMMA-modified PTAA induces (111) preferred orientation growth of perovskite for higher-quality films.

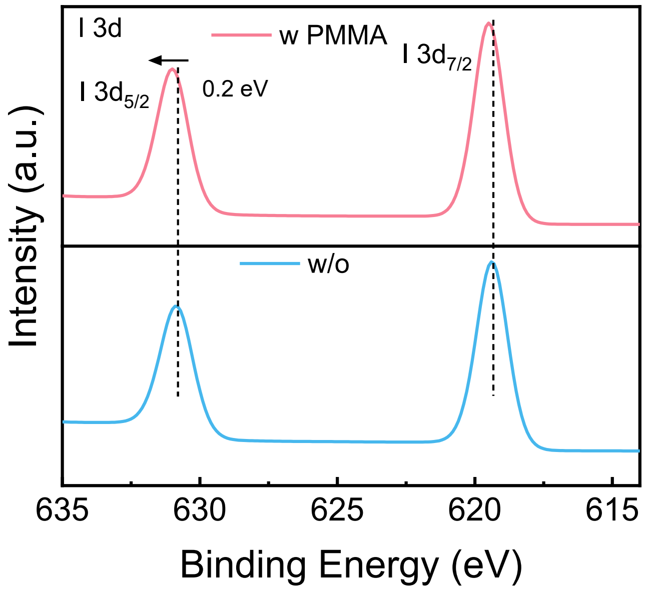

Figure S8. The X-ray photoelectron spectroscopy (XPS) spectra of the I 3d orbitals in perovskite films without and with PMMA.

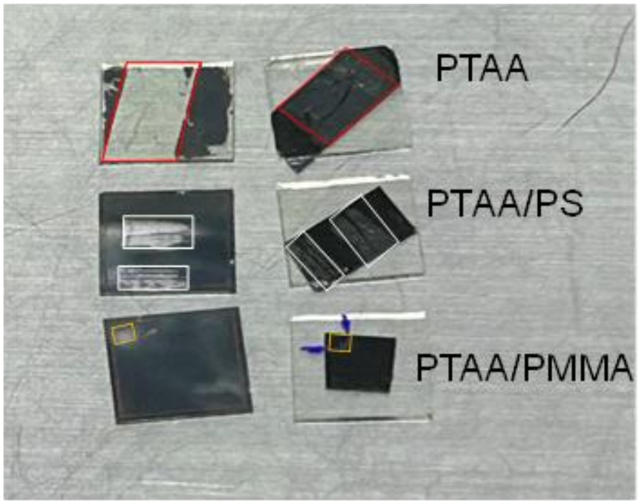

Figure S9. Various revealed perovskites on PTAA, PTAA/PS and PTAA/PMMA visualized by an Optical camera. The samples modified with PMMA could hardly separate the perovskite from the bottom PTAA interface, indicating the enhanced interface bonding force by PMMA modification. After being modified with PMMA or PS, the interface bonding force between PTAA and perovskite was enhanced. When we peeled off the bottom interface with tape, we found that the samples modified with PMMA could hardly separate the perovskite from the bottom PTAA interface. It is only can peel off a tiny area of the perovskite film for the PMMA modification sample.

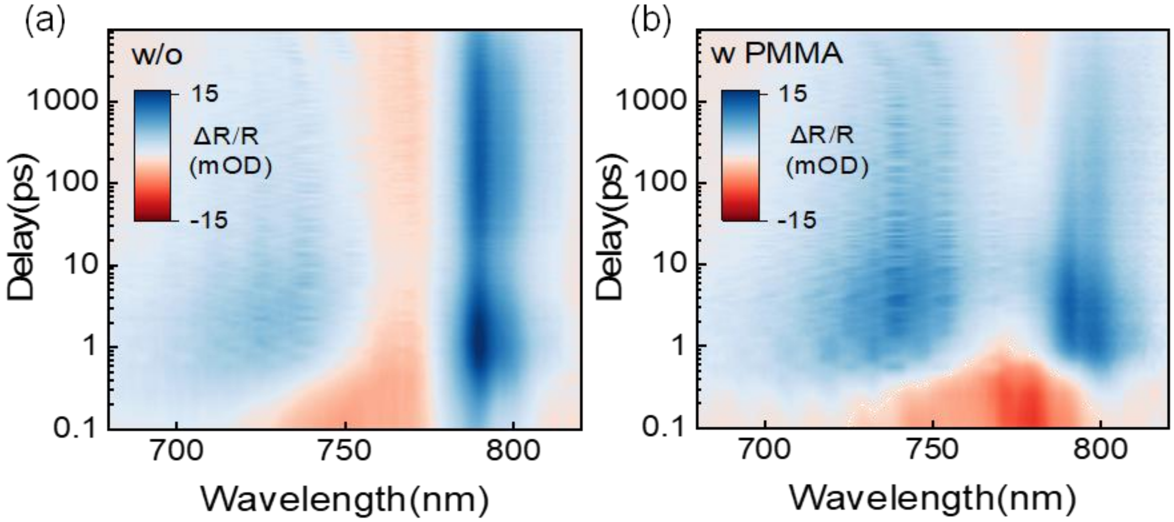

Figure S10. The transient reflection (TR) spectroscopy of the perovskite films without and with PMMA.

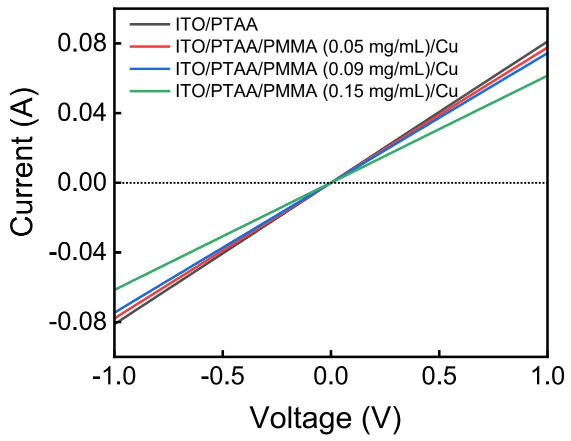

Figure S11. Current-voltage (*I*-*V*) characteristics of (a) ITO/PTAA/Au and (b) ITO/PTAA/PMMA/Au devices with various PMMA concentration.

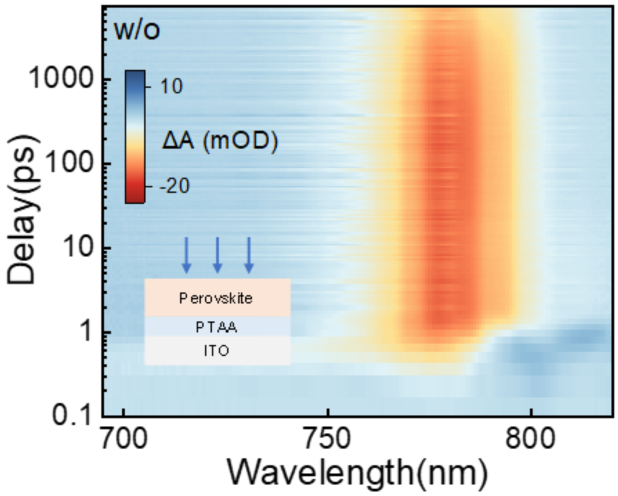

Figure S12. The ultrafast transient absorption (TA) spectroscopy of the control perovskite films.

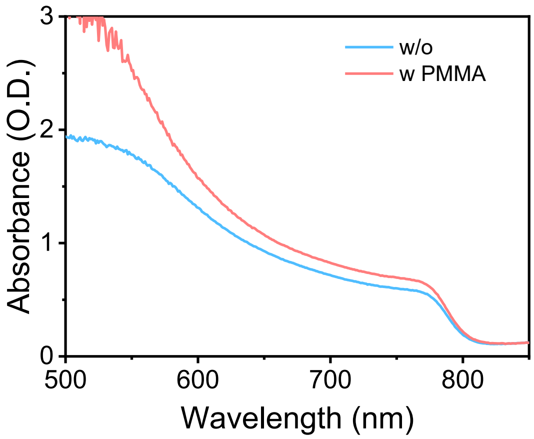

Figure S13. The absorption spectra of perovskite films without (control) and with the UNLC of PMMA. The increase of light absorption intensity indicates that the film has higher crystallinity, and no obvious change of absorption band edge indicates that PMMA modification does not change the composition of the film.

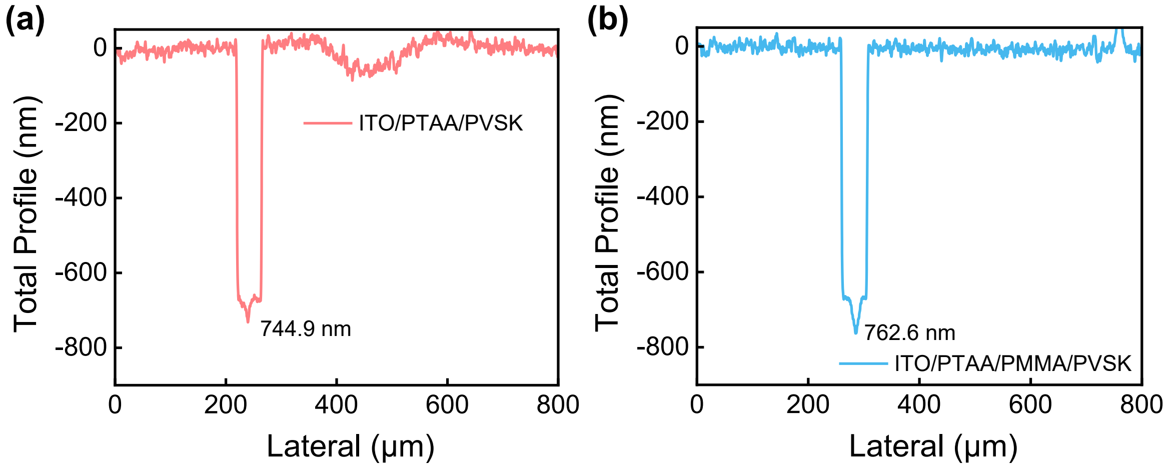

Figure S14. The perovskite films thickness deposited on PTAA layers (a) without or (b) with PMMA-modification, which is tested by the step profiler.

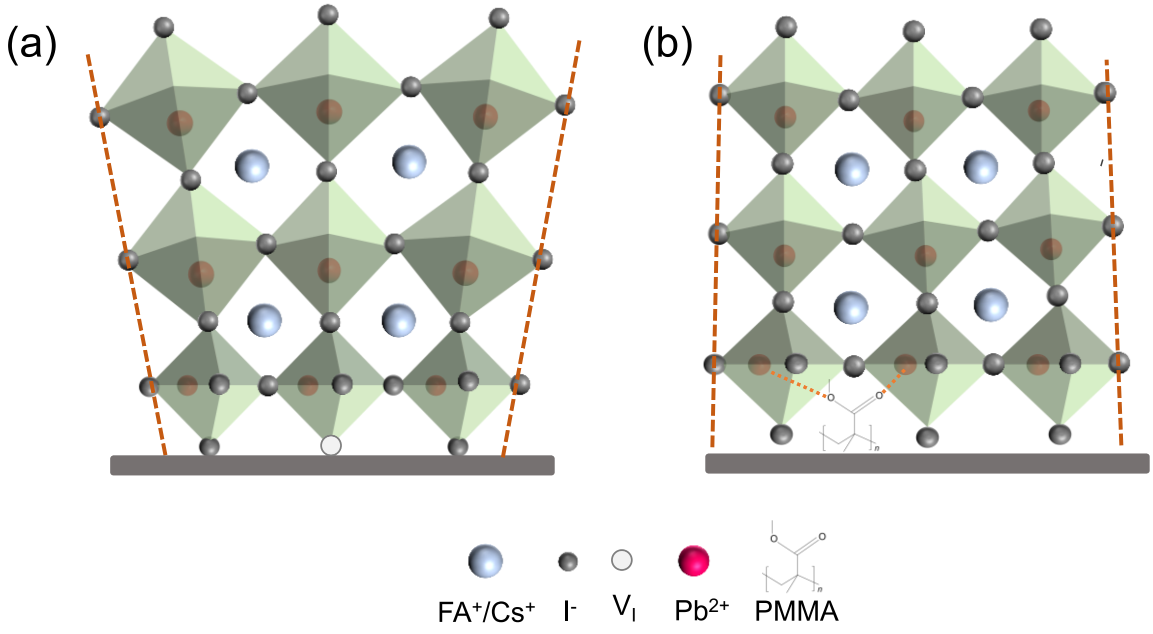

Figure S15. The schematic diagram of the strain release for control and PMMA-modified films. It regulates the tension strain to be compressive strain by the bottom surface modification with PMMA.

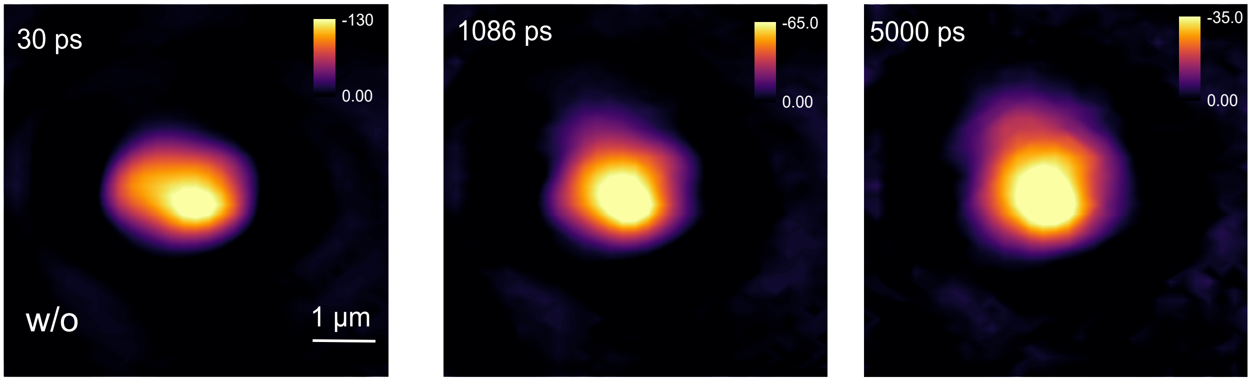

Figure S16. The transient absorption microscopy imaging of carrier transport in the $FA_{0.90}Cs_{0.10}PbI_{2.93}Br_{0.17}$-based perovskite films without the UNLC of PMMA.

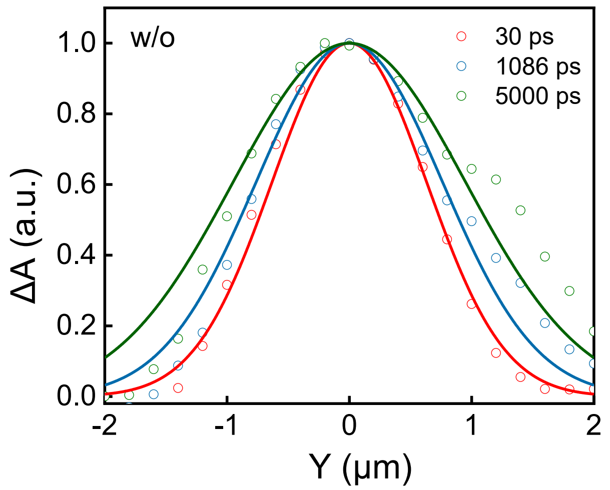

Figure S17. Scans of excited-state density profiles projected along Y axis as the delay times for $FA_{0.90}Cs_{0.10}PbI_{2.93}Br_{0.17}$-based film, which are fitted with Gaussian functions.

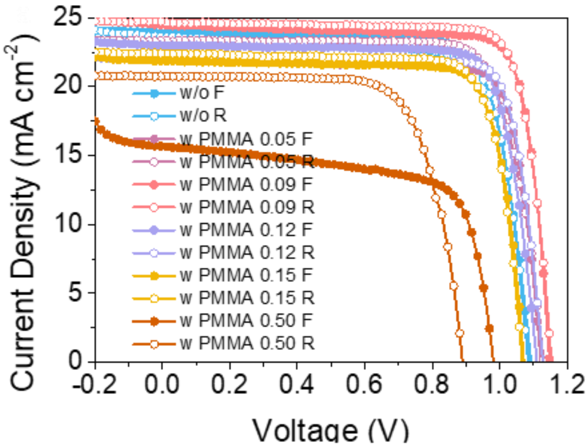

Figure S18. Current density–voltage (*J*–*V*) curves for the champion control and PMMA PSCs with various concentrations measured under reverse scan (RS) and forward scan (FS).

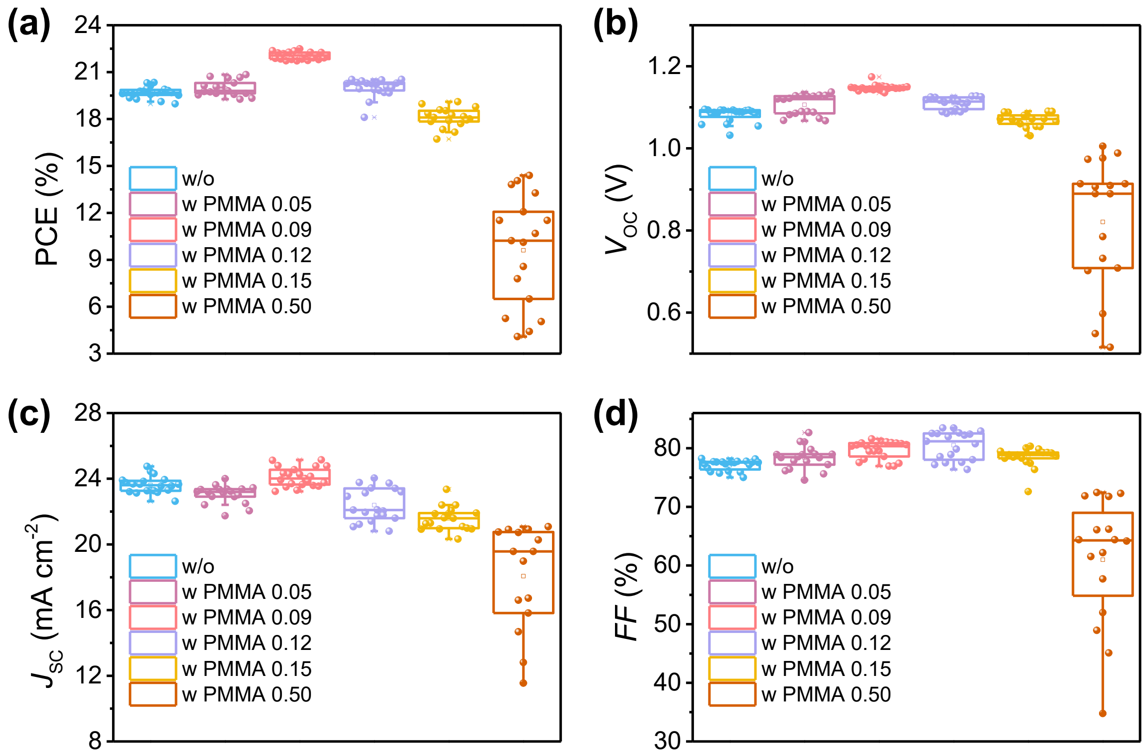

Figure S19. Statics of (a) V_{OC} and (b) *FF* for over 20 devices without or with PMMA modification. In a typical box plot, the line in the box is the median line, and the square in the center of the box is the average point.

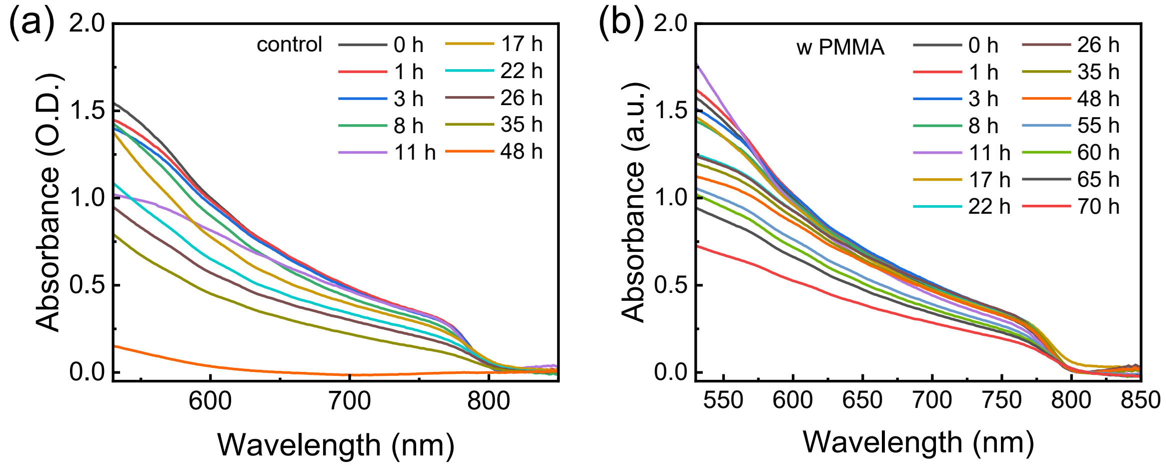

Figure S20. Absorption spectra of perovskite films with and without the UNLC of PMMA on PTAA aged in pure oxygen atmosphere combined with illumination (LED, 1 sun). The test of light and oxygen stability of perovskite films was carried out under a combination of pure oxygen (10^5 Pa) and white light (100 mW cm^{-2}) treatment.

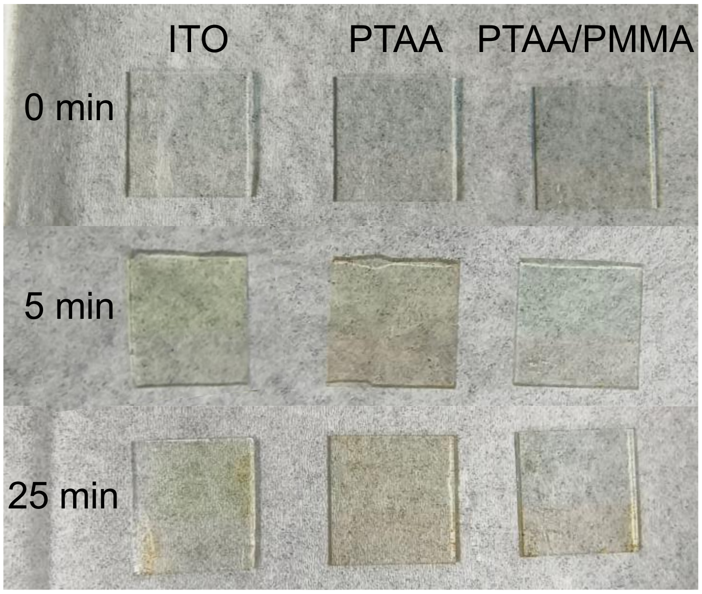

Figure S21. Photographs of the reactions at different times of 5 min and 25 min between PTAA films without and with PMMA and I_2 vapor under 50 °C annealing. The results demonstrate that I_2 can permeate into PTAA and react with it, the PMMA can alleviate this reaction.

Supplementary Table S1. The fitted TRPL (time-resolved photoluminescence) decay lifetimes of $FA_{0.90}Cs_{0.10}PbI_{2.93}Br_{0.17}$-based PSCs without or with PMMA modification.

**$FA_{0.90}Cs_{0.10}PbI_{2.93}Br_{0.17}$**	A_1	τ_1 (ns)	A_1	τ_2 (ns)	τ_{ave} (ns)
w/o	0.50	13.15	0.50	19.31	68
w PMMA	0.67	6.18	0.32	41.55	33

The spectra are fitted by the biexponential equation: $Y = A_1exp(-t/\tau_1) + A_2exp(-t/\tau_2)$, where τ_1 and τ_2 denote the fast and slow decay time constants, relating to the radiative and trap-assisted nonradiative recombination processes, respectively.

Supplementary Table S2. Photovoltaic parameters of the $FA_{0.90}Cs_{0.10}PbI_{2.93}Br_{0.17}$-based PSCs without or with PMMA modification.

**$FA_{0.90}Cs_{0.10}PbI_{2.93}Br_{0.17}$**	Scanning direction	V_{OC} (V)	J_{SC} (mA cm^{-2})	FF (%)	PCE (%)
w/o	R	1.09	23.94	76.7	20.3
w/o	F	1.08	23.68	77.2	19.8
w PMMA 0.05	R	1.13	23.34	78.4	20.7
w PMMA 0.05	F	1.12	22.15	81.1	20.1
w PMMA 0.09	R	1.15	24.21	81.2	22.6
w PMMA 0.09	F	1.14	24.50	80.2	22.4
w PMMA 0.12	R	1.13	22.10	82.3	20.5
w PMMA 0.12	F	1.11	23.03	79.9	20.4
w PMMA 0.15	R	1.07	21.91	80.0	18.8
w PMMA 0.15	F	1.07	22.30	79.5	18.9
w PMMA 0.50	R	0.89	20.73	71.8	13.2
w PMMA 0.50	F	0.98	15.75	68.5	10.6

Supplementary Table S3. The transition voltage (V_{tr}) from the linear transport to the trap-filling current region, the density of traps (N_{trap}), and the charge carrier mobility (μ) were evaluated by

the space-charge-limited current (SCLC) technique.

$FA_{0.90}Cs_{0.10}PbI_{2.93}Br_{0.17}$	w/o	w
	hole	hole
V_{tr} [V]	0.538	0.082
N_{trap} [cm^{-3}]	3.47×10^{15}	5.29×10^{14}
μ [cm^2 V^{-1} s^{-1}]	7.42×10^{-4}	6.01×10^{-3}
